# Knowledge, attitudes and practices of health personnel of maternities in the prevention of mother-to-child transmission of HIV in a sub-Saharan African region with high transmission rate: some solutions proposed

**DOI:** 10.1186/s12884-018-1876-0

**Published:** 2018-06-14

**Authors:** Elie Nkwabong, Romuald Meboulou Nguel, Nelly Kamgaing, Anne Sylvie Keddi Jippe

**Affiliations:** 1 Department of Obstetrics & Gynecology, University Teaching Hospital & Faculty of Medicine and Biomedical Sciences, University of Yaoundé I, Yaoundé, Cameroon; 2 Faculty of Medicine and Biomedical Sciences, University of Yaoundé I, Yaoundé, Cameroon; 3 Department of Pediatrics, Faculty of Medicine and Biomedical Sciences, University of Yaoundé I, Yaoundé, Cameroon; 4 Department of Epidemiology, Faculty of Medicine and Biomedical Sciences, University of Yaoundé I, Yaoundé, Cameroon

**Keywords:** Health personnel, Mother-to-child transmission of HIV, Knowledge, Attitude and practices for the prevention of HIV transmission

## Abstract

**Background:**

High mother-to-child (MTC) transmission rate of HIV might contribute to the increased pandemic rate. The aim of this study was to identify the knowledge, attitude and practices of health personnel working in maternities in the prevention of MTC transmission of HIV.

**Methods:**

This cross-sectional descriptive study was carried out from 20th February to 30th April, 2017. All health personnel working in the maternity wards were included in this study. The variables recorded included their age, grade, experience (number of year of practice), gender, educational level, health structure and the training in prevention of MTC transmission of HIV. Analyses were done using SPSS 21.0. The Pearson Chi-square test or Fisher’s exact test and logistic regression were used for comparison. The level of significance was *P* < 0.05.

**Results:**

A total of 140 health personnel were recruited. Knowledge was insufficient amongst 73 of them (52.1%). The factors significantly associated with sufficient knowledge were midwifery qualification (aOR 9.01, 95% CI 1.82–48.60) and training in prevention of MTC transmission of HIV (aOR 2.23, 95% CI 1.02–4.81). Regarding attitudes, it was negative in 85 practitioners (60.7%). Only those aged ≥33 years were significantly associated with a positive attitude (aOR 2.34, 95% CI 1.14–4.23). As concerns practices, only 32 practitioners (22.9%) had good practices. Only midwives were associated with good practices (aOR 3.23, 95% CI 1.21–9.95).

**Conclusion:**

Insufficient knowledge, attitude and practices in the prevention of MTC transmission of HIV were observed among the majority of health personnel in the region. This lack of knowledge in prevention can therefore contribute to the rise of the mother-to-child transmission rate of HIV. To reduce this rate, more health personnel should be trained, especially midwives, in the prevention of MTC transmission of HIV. Moreover, deliveries of all women living with HIV should be conducted or at least supervised by trained midwives, especially those of at least 33 years of age.

**Electronic supplementary material:**

The online version of this article (10.1186/s12884-018-1876-0) contains supplementary material, which is available to authorized users.

## Background

Globally in 2015, an estimated 38.8 million people were living with HIV [1]. The use of antiretroviral drugs (ARV) has considerably reduced the mortality from HIV infection, at least in some countries [2, 3]. This reduced mortality rate observed since 2005 is associated with an increased life expectancy which can contribute to the increase of the pandemic rate [1, 4]. The HIV pandemic rate might continue to rise due to persistence of the mother-to-child (MTC) transmission [5]. This mode of transmission is responsible for more than 90% of HIV infections in children. It is estimated that almost 400,000 newborns are infected with HIV through mother-to-child transmission every year [6]. Sexually active women in their reproductive age are more prone to HIV infection [7]. Therefore, more women living with HIV are expected to be received in the maternity units.

The MTC transmission rate has significantly reduced to less than 1% in high income countries [6]. This reduction is attributed to the systematic use of ARV by pregnant women living with HIV, the avoidance of amniocentesis, fetal membranes stripping, artificial rupture of membranes, fetal scalp puncture, episiotomy, instrumental delivery, milking of the umbilical cord prior to its section, systematic airways aspiration. Other compulsory measures for the reduction of the MTC transmission rate include systematic cleaning of the umbilical cord with an antiseptic prior to its section, systematic bathing of the newborn prior to injection of vitamin K and administration of ARV to the newborn. Also, artificial feeding should be preferred to breastfeeding [8–10]. The persistence of MTC transmission of HIV in some high-income countries is due to late diagnosis of HIV with late onset of ARV drugs [5].

In some low-income countries, the MTC transmission rate of HIV still remains as high as 8.9% [8]. Neonatal HIV infection can be avoided, if not minimized, if all the recommendations are observed. The HIV prevalence amongst pregnant women in Cameroon was 7.8% in 2011, but the Eastern region had one of the highest recorded rates (9.1%) [11]. The national MTC transmission rate in the country was 9.6% in 2009 [12]. Stigmatization and discrimination practiced by some health personnel, still remain a major problem in some parts of Cameroon. Moreover, some recommendations for the prevention of MTC transmission of HIV might not be observed by all.

The high MTC transmission rate might partly be due to the non-observance of some of the recommendations listed above. The aim of this study was to assess the knowledge, attitudes and practices of health personnel on the prevention of MTC transmission of HIV in order to find out the factors that can influence this high MTC transmission rate.

## Methods

This cross-sectional descriptive study was carried out between 20th February and 30th April, 2017. All health personnel of the 54 maternities of the region (seven hospitals and 47 health centers) who assist women during deliveries took part in this survey. Less experienced staff members (˂1-year experience) were excluded as well as those who refused to participate in this study. A written informed consent was obtained from each participant. This study was approved by the institutional ethics committee.

In our country, pregnant women screened positive to HIV receive from a trained nurse or doctor ARV according to the actual national protocol (Tenofovir, Lamivudine and Efavirenz as first line treatment). This health care provider also counsels these women on various items including hygiene, nutrition and sexual intercourse. In cases of side effects or viral resistance to these drugs, the patient is sent to a trained medical doctor who can administer other alternative regimens. The pregnancy is then regularly followed up. Women are counseled to report to the labor ward as soon as uterine contractions have started, in cases of premature rupture of membranes or any other complaints.

The variables recorded on an auto-administered questionnaire by the principal investigator included participant’s age and sex, grade, health structure, number of years of experience, eventuality of training in the prevention of MCT transmission of HIV. Moreover, to evaluate knowledge, attitudes and practices to reduce the MTC transmission of HIV, 10 other questions were asked in each section.

The 10 questions for knowledge were as follows: can a mother transmit HIV to her baby? When does this transmission occur? When is this transmission highest? When should highly active antiretroviral therapy (HAART) be started? What is the protocol actually recommended by the country? Should artificial rupture of membranes be done during labor? How should newborn ideally be fed? What drug is given to newborn to prevent MTC transmission of HIV? For how long should that drug be given? If the mother took HAART for less than four weeks, for how long should the newborn take this medication? A minimum score of eight correct answers out of 10 reflected sufficient knowledge while less than 8 correct answers reflected insufficient knowledge.

The 10 questions for attitudes were: Are you at ease when receiving a woman infected with HIV? Do you observe confidentiality? Do you obtain informed consent before testing for HIV? Is your practice influenced by the fact that you can be contaminated? Do you avoid stigmatization? Do you avoid discrimination? Do you need special motivation to take care of the infected women? Do you need special material to protect yourself? Are you interested in the newborn’s protection? Will you like to continue to take care of HIV infected women? A minimum of eight correct answers out of 10 reflected positive attitudes while less than eight correct answers reflected negative attitudes.

The 10 questions for practice were as follows: What do you do if the contractions are weak? What do you do in case of premature rupture of membranes lasting more than four hours? How frequently do you perform digital vaginal examination? Do you conduct artificial rupture of membranes? Do you disinfect the umbilical cord prior to its section? Do you milk the umbilical cord prior to its section? Do you systematically aspirate the newborns airways? Do you bathe the newborn with an antiseptic solution? Do you administer nevirapine to the newborn within the first 48 first hours? Do you screen all parturients with unknown HIV status? A minimum of eight correct answers out of 10 reflected good practices, while less than eight correct answers reflected poor practices.

Our minimum sample size of 120 participants was calculated using the following formula N=P×(1-P) × (Zα/D)^2^ [13] where P is the percentage of participants with good knowledge in an African study (8.5%) [14], Zα = 1.96 corresponding to a type I error of 5% and D = 0.05 is the degree of precision.

The data were analyzed using the SPSS 21.0. Data of women with insufficient knowledge, negative attitudes and poor practices were compared to those of women with sufficient knowledge, positive attitudes and good practices respectively. The Pearson Chi-square test or the fisher’s exact test were used for comparison. Logistic regression analysis was undertaken to control for potential confounders. *P* < 0.05 was considered statistically significant.

## Results

A number of 178 health personnel were interviewed, 38 were excluded either because they found the questionnaire difficult, hence refused to participate (17 cases), they had less than one year experience (9 cases) or they wanted to fill the form at home and to return it two to four days later (12 cases). The remaining 140 participated in this survey. Their ages ranged from 23 to 54 years (mean 34.3 ± 7.9). Concerning gender, 101 (72.1%) were females as against 39 males (27.9%).

Regarding their qualification, 54 (38.6%) were nurse aides, 32 (22.8%) assistant nurses, 35 (25%) state-registered nurses, 14 (10%) midwives and five (3.6%) health technicians. These personnel were working in various hospitals (40 or 28.6%) and in health centers (100 or 71.4%).

With regards to the number of years of experience in the maternity, it ranged from 1 to 29 years (mean 5.4 ± 5.9). A total of 76 (54.3%) were trained in the prevention of MCT transmission of HIV while the remainder (64 or 45.7%) were trained in their daily practice by the trained ones.

Of the 140 personnel, knowledge of 67 (47.9%) was sufficient. It was insufficient amongst 73 of them (52.1%). For instance, only 42.9% (60 participants) knew that MTC transmission is highest during labor. Table 1 summarizes knowledge distribution in relation to some variables. Additional file 1 shows details of answers according to grade of participants. After logistic regression, the factors significantly associated with sufficient knowledge were midwifery qualification (aOR 9.01, 95% CI 1.82–48.60, *P* = 0.008) and formal training in the prevention of MTC transmission of HIV (aOR 2.23, 95% CI 1.02–4.81, *P* = 0.048).

**Table 1:** Knowledge distribution among the population under study

Variables		Sufficient knowledge N (%)	Insufficient knowledge N (%)	OR	95% CI	*P*-value
Age (year)	≥33 (*n* = 61)	35 (57.4)	26 (42.6)	1.97	1.00–3.89	0.047
	˂33 (*n* = 79)	32 (40.5)	47 (59.5)			
Sex	Female (*n* = 101)	52 (51.5)	49 (48.5)	1.69	0.79–3.60	0.168
	Male (*n* = 39)	15 (38.5)	24 (61.5)			
Qualification	Midwives (*n* = 14)	12 (85.7)	2 (14.3)	7.74	1.66–36.05	0.009
	Others (*n* = 126)	55 (43.6)	71 (56.3)			
Health structure	Hospital (*n* = 40)	22 (55)	18 (45)	1.49	0.71–3.12	0.285
	Health centers (*n* = 100)	45 (45)	55 (55)			
Experience (year)	≥6 (*n* = 41)	24 (58.5)	17 (41.5)	1.83	0.87–3.84	0.105
	≤5 (*n* = 99)	43 (43.4)	56 (56.6)			
Training in PMTCT	In seminars (*n* = 76)	47 (61.9)	29 (38.1)	3.56	1.76–7.19	0.0004
	By senior colleagues (*n* = 64)	20 (31.2)	44 (68.8)			

*CI* Confidence Interval, *OR* Odds Ratio, *PMTCT* Prevention of mother-to-child transmission

Regarding attitudes, it was negative in 85 practitioners (60.7%). It was positive only in 55 (39.3%). For instance, 112 participants (80%) were not at ease when taking care of a pregnant woman living with HIV. The distribution of attitudes is shown in Table 2. Detailed answers according to grade of participants are given in additional file 2. Only age ≥ 33 years was significantly associated with a positive attitude after logistic regression (aOR 2.34, 95% CI 1.14–4.23, *P* = 0.030).

**Table 2:** Attitude distribution among the population under study

Variables		Positive attitude	Negative attitude	OR	95% CI	*P*-value
Age (year)	≥33 (n = 61)	30 (49.2)	31 (50.8)	2.09	1.04–4.16	0.036
	˂33 (n = 79)	25 (31.6)	54 (68.4)			
Sex	Male (n = 39)	19 (48.7)	20 (51.3)	1.71	0.81–3.62	0.157
	Female (n = 101)	36 (35.6)	65 (64.4)			
Qualification	AN (*n* = 32)	19 (59.3)	13 (40.7)	2.92	1.29–6.57	0.009
	Others (*n* = 108)	36 (33.3)	72 (66.7)			
Health structure	Hospital (n = 40)	20 (50)	20 (50)	1.85	0.88–3.90	0.102
	Health centers (n = 100)	35 (35.0)	65 (65.0)			
Experience (year)	≥6 (n = 41)	14 (34.1)	27 (65.9)	0.73	0.34–1.56	0.423
	≤5 (n = 99)	41 (41.4)	58 (58.6)			
Training in PMTCT	Seminars (n = 76)	29 (38.2)	47 (61.8)	0.90	0.45–1.78	0.765
	Other colleagues (n = 64)	26 (40.6)	38 (59.4)			

*CI* Confidence Interval, *OR* Odds Ratio, *AN* Assistant Nurse, *PMTCT* Prevention of mother-to-child transmission

As concerns practices, only 32 practitioners (22.9%) had good practices as against 108 (77.1%) with poor practices. Indeed, 101 participants (72.1%) did not know that labor should be augmented in cases of failure to progress. Its distribution in relation to some variables is given in Table 3. Additional file 3 provides details of answers according to grade of participants. After logistic regression, only midwives were associated with good practices (aOR 3.23, 95% CI 1.21–9.94, *P* = 0.041).

**Table 3:** Practice distribution among the population under study

Variables		Good practice	Poor practice	OR	95% CI	*P*-value
Age (year)	≥33 (n = 61)	14 (23.0)	47 (77.0)	1.00	0.45–2.23	0.981
	˂33 (n = 79)	18 (22.8)	61 (77.2)			
Sex	Female (n = 101)	28 (27.7)	73 (72.3)	3.35	1.09–10.31	0.034
	Male (n = 39)	4 (10.3)	35 (89.7)			
Qualification	Midwives (n = 14)	7 (50)	7 (50)	4.04	1.29–12.57	0.015
	Others (n = 126)	25 (19.8)	101 (80.2)			
Health structure	Hospital (n = 40)	20 (50)	20 (50)	3.54	1.62–7.73	0.001
	Health centers (n = 100)	22 (22)	78 (78)			
Experience (year)	≤5 (n = 99)	23 (23.2)	76 (76.8)	1.07	0.44–2.57	0.869
	≥6 (n = 41)	9 (21.9)	32 (78.1)			
Training in PMTCT	Seminars (n = 76)	18 (23.7)	58 (76.3)	1.10	0.50–2.45	0.799
	Other colleagues (n = 64)	14 (21.9)	50 (78.1)			

*CI* Confidence Interval, *OR* Odds Ratio, *PMTCT* Prevention of mother-to-child transmission

Table 4 summarizes the factors associated with sufficient knowledge, positive attitude and good practices.

**Table 4:** Factors associated with sufficient knowledge, positive attitude and good practice

Variables		OR	95% CI	*P*-value	aOR	95% CI	*P*-value
Sufficient knowledge	Midwife qualification	7.74	1.66–36.05	0.009	9.01	1.82–48.60	0.008
	Training in PMTCT	3.56	1.76–7.19	0.0004	2.23	1.02–4.81	0.048
	Age ≥ 33 years	1.97	1.00–3.89	0.047	1.20	0.94–1.86	0.141
Positive attitude	Age ≥ 33 years	2.09	1.04–4.16	0.036	2.34	1.14–4.23	0.030
	Assistant Nurse	2. 92	1.29–6.57	0.009	1.55	0.87–1.92	0.210
Good practice	Midwife qualification	4.04	1.29–12.57	0.015	3.23	1.21–9.95	0.041
	Practicing in Hospitals	3.54	1.62–7.73	0.001	1.73	0.84–2.12	0.079
	Female sex	3.35	1.09–10.31	0.034	1.60	0.70–2.34	0.089

*CI* Confidence Interval, *OR* Odds Ratio, *aOR* adjusted Odds Ratio, *PMTCT* Prevention of mother-to-child transmission

Knowledge was significantly associated with attitude (*P* = 0.02), attitude with practice (P = 0.02), but knowledge was insignificantly associated with practice (*P* = 0.3).

## Discussion

Our study was carried out in an area where the majority of health structures, especially the health centers, are poorly equipped with only few midwives available. The mean age of participant in our study (34.3 years) was lower than that of 40.9 observed in South Africa. The proportion of females in our study (72.1%) was lower than the 88.6 to 94.2% observed in South Africa [15]. The mean years of experience in this study (5.4 years) seemed small. This is due to the fact that for many years, retired health personnel were not replaced by the ministry of public health due to financial constraints. It is only recently that younger health professionals are being recruited. Their number is still very small. Efforts should be made by government to increase the number of health personnel not only in this region, but also in the entire country. Due to scarcity of midwives, many other health personnel had to assist women during delivery.

Daily, studies are being carried out on the HIV pandemic worldwide. New recommendations have to be shared with other health providers. Because of financial constraints, only 54.3% of health personnel of the study population participated in the training on guidelines in the prevention of MTC transmission of HIV. More trainings should be offered to health professionals, each time there are new recommendations.

We observed that the majority of health personnel had insufficient knowledge even though our questions were simple. Poor knowledge was already observed in some African countries [15, 16]. This can be explained in our study by the smaller number of years of experience (mean 5.4 years), by the fact that the majority of personnel were not midwives and the absence of training in prevention of MTC transmission of HIV. Indeed, after logistic regression, the only factors associated with sufficient knowledge were midwifery qualification and training in the prevention of MTC transmission of HIV. This shows that to improve the knowledge required in the prevention of vertical transmission of HIV, more midwives should be trained and posted in all the maternities of this region. Similar results could be found in other settings in the country given that there is scarcity of trained health personnel in the whole country.

Also, while waiting for midwives to be trained in sufficient numbers, the government should look for funding necessary to train other health personnel in the prevention of MTC transmission of HIV. In other studies, better knowledge was observed amongst registered nurses with previous HIV/AIDS training [15]. In our survey, practitioners with more than five-year experience had better knowledge but the difference was insignificant (*P* = 0.10). Other researchers noticed that neither age, gender, professional experience, nor health structure influenced knowledge, as found in our study [16].

Regarding attitudes, the majority had negative attitudes (50.8%) (Table 2), as already observed in a study in Nigeria [16]. This was in contrast with the findings of some authors where practitioners had mainly positive attitudes [15]. After logistic analysis, only heath personnel of at least 33 years old had positive attitudes. Younger ones might consider women living with HIV as responsible for their infection or as contagious to health personnel. The health personnel, especially the younger, should be taught that negative attitudes might lead to poor practice, and therefore to increased risk of MTC transmission.

With regards to practice, after logistic regression, good practices were observed mainly amongst midwives. This can be explained by the fact that during their training they are taught labor management at different stages, neonatal care as well as how to deliver HIV infected women. Other researchers observed good practices amongst registered nurses of the maternity wards [15]. Although good knowledge was observed mainly amongst midwives, only 50% of midwives in our survey had good practices (Table 3). Some of them were complaining of the lack of motivation, protection and supervision. Therefore, midwives also should be trained in the prevention of MTC transmission of HIV and motivated.

Sufficient knowledge, positive attitudes and good practices are needed in the prevention of the vertical transmission of HIV. More midwives should be trained with emphasis on the prevention of MTC transmission of HIV and on the attitudes to be adopted towards HIV infected women.

The limitations of our study are our inability to be certain of the veracity of some answers given. Also, we did not evaluate the accessibility to HIV testing and access to care. Furthermore, we did not evaluate neither ARV administration and timing of medication nor the mode of newborn feeding in this region as they also influence the MTC transmission rate. More studies should be conducted among women living with HIV and/or their offspring to evaluate the other factors that might explain high MTC transmission of HIV.

## Conclusion

This study found insufficient knowledge, attitude and practices of health personnel in the prevention of MTC transmission of HIV in the region. This lack of knowledge in prevention can partly explain the high mother-to-child transmission rate of HIV. Henceforth, the government should look for funding necessary to train more health personnel, especially midwives, specifically in the prevention of MTC transmission of HIV. Moreover, deliveries of all women living with HIV should be conducted, or at least supervised by midwives, especially those aged at least 33 years.

## Additional files

Additional file 1: **Table S1.** Knowledge distribution according to grade of participants. Contains details of answers assessing knowledge according to grade of participants as well as the statistical analysis. (DOC 60 kb)

Additional file 2: **Table S2.** Attitude distribution according to grade of participants. Contains details of answers assessing attitude according to grade of participants as well as the statistical analysis. (DOC 58 kb)

Additional file 3: **Table S3.** Practice distribution according to grade of participants. Contains details of answers assessing practice according to grade of participants as well as the statistical analysis. (DOC 56 kb)

## Abbreviations

AIDS: Acquired human Immunodeficiency Syndrome
AN: Assistant Nurse
aOR: adjusted Odds Ratio
ARV: Antiretroviral drugs
CI: Confidence Interval
HAART: Highly active antiretroviral therapy
HIV: Human immunodeficiency virus
MTC: Mother-to-child
OR: Odds Ratio
PMTCT: Prevention of mother-to-child transmission
