# Supplementary material for: Knowledge, attitudes and practices of health personnel of maternities in the prevention of mother-to-child transmission of HIV in a sub-Saharan African region with high transmission rate: some solutions proposed
Source: BMC Pregnancy Childbirth. 2018 Jun 14;18:227. doi: 10.1186/s12884-018-1876-0 (PMC6000955; doi:10.1186/s12884-018-1876-0)
Supplement: Supplementary file 1 — Table S1. Knowledge distribution according to grade of participants. Contains details of answers assessing knowledge according to grade of participants as well as the statistical analysis. (DOC 60 kb) [file 12884_2018_1876_MOESM1_ESM.doc]

**Additional file 1: Table S1.** Knowledge distribution according to grade of participants.

| Questions Answer | | Grade of participants | | | | | | | |
| --- | --- | --- | --- | --- | --- | --- | --- | --- | --- |
|  |  | Total  N(%) | NA  N (%) | AN  N (%) | SRN  N (%) | MW  N (%) | HT  N (%) |  | P |
|  | |  |  |  |  |  |  |  |  |
| 1. Can a mother transmit HIV to her baby? | WA | 4(2.9) | 3(5.6) | 0(0.0) | 0(0.0) | 0(0.0) | 1(20.0) |  | 0.1 |
|  | CA | 136(97.1) | 51(94.4) | 32(100) | 35(100) | 14(100) | 4(80.0) |  |  |
| 2. When does this transmission occur? | WA | 54(38.6) | 27(50.0) | 11(34.4) | 10(28.6) | 1(7.1) | 5(100) |  | 0.001 |
|  | CA | 86(61.4) | 27(50.0) | 21(65.6) | 25(71.4) | 13(92.9) | 0(0.0) |  |  |
| 3. When is this transmission highest? | WA | 60(42.9) | 29(53.7) | 16(50.0) | 10(28.6) | 3(21.4) | 2(40.0) |  | 0.08 |
|  | CA | 80(57.1) | 25(46.3) | 16(50.0) | 25(71.4) | 11(78.6) | 3(60.0) |  |  |
| 4. When should highly active antiretroviral therapy (HAART) be started? | WA | 9(6.4) | 6(11.1) | 1(3.1) | 0(0.0) | 1(7.1) | 1(20.0) |  | 0.2 |
|  | CA | 131(93.6) | 48(88.9) | 31(96.9) | 35(100) | 13(92.9) | 4(80.0) |  |  |
| 5. What is the protocol actually recommended by the country? | WA | 14(10.0) | 8(14.8) | 2(6.3) | 2(5.7) | 1(7.1) | 1(20.0) |  | 0.3 |
|  | CA | 126(90,0) | 46(85,2) | 30(93,8) | 33(94.3) | 13(92,9) | 4(80,0) |  |  |
| 6. Should artificial rupture of membranes be done during labor? | WA | 66(47.1) | 32(59.3) | 10(31.3) | 17(48.6) | 2(14.3) | 5(100) |  | 0.002 |
|  | CA | 74(52.9) | 22(40.7) | 22(68.8) | 18(51.4) | 12(85.7) | 0(0.0) |  |  |
| 7. How should newborn ideally be fed? | WA | 42(30.0) | 22(40.7) | 5(15.6) | 8(22.9) | 4(28.6) | 3(60.0) |  | 0.07 |
|  | CA | 98(70.0) | 32(59.3) | 27(84.4) | 27(77.1) | 10(71.4) | 2(40.0) |  |  |
| 8. What drug is given to newborn to prevent MTC transmission of HIV? | WA | 5(3.6) | 2(3.7) | 0(0.0) | 1(2.9) | 1(7.1) | 1(20.0) |  | 0.3 |
|  | CA | 135(96.4) | 52(96.3) | 32(100) | 34(97.1) | 13(92.9) | 4(80.0) |  |  |
| 9. For how long should that drug be given? | WA | 79(56.4) | 29(53.7) | 19(59.4) | 19(54.3) | 8(57.1) | 4(80.0) |  | 0.9 |
|  | CA | 61(43.6) | 25(46.3) | 13(40.6) | 16(45.7) | 6(42.9) | 1(20.0) |  |  |
| 10. If the mother took HAART for less than four weeks, for how long should the newborn take this medication? | WA | 44(31.4) | 13(24.1) | 9(28.1) | 16(45.7) | 3(21.4) | 3(60.0) |  | 0.2 |
|  | CA | 96(68.6) | 41(75.9) | 23(71.9) | 19(54.3) | 11(78.6) | 2(40.0) |  |  |

NA: Nurse aide, AN: Assistant nurse, SRN: State-registered nurse, MW: Midwife, HT: Health technician, CA: Correct answer, WA: Wrong answer, HAART: Highly active antiretroviral therapy, MTC: Mother-to-child.
