# Supplementary material for: Knowledge, attitudes and practices of health personnel of maternities in the prevention of mother-to-child transmission of HIV in a sub-Saharan African region with high transmission rate: some solutions proposed
Source: BMC Pregnancy Childbirth. 2018 Jun 14;18:227. doi: 10.1186/s12884-018-1876-0 (PMC6000955; doi:10.1186/s12884-018-1876-0)
Supplement: Supplementary file 2 — Table S2. Attitude distribution according to grade of participants. Contains details of answers assessing attitude according to grade of participants as well as the statistical analysis. (DOC 58 kb) [file 12884_2018_1876_MOESM2_ESM.doc]

**Additional file 2: Table S2.** Attitude distribution according to grade of participants.

| Questions Answer | | Grade of participants | | | | | | | |
| --- | --- | --- | --- | --- | --- | --- | --- | --- | --- |
|  |  | Total  N(%) | NA  N(%) | AN  N(%) | SRN  N(%) | MW  N(%) | HT  N(%) |  | P |
| 1. Are you at ease when receiving a woman living with HIV? | WA | 28(20.0) | 16(29.6) | 5(15.6) | 6(17.1) | 1(7.1) | 0(0.0) |  | 0.2 |
|  | CA | 112(80.0) | 38(70.4) | 27(84.4) | 29(82.9) | 13(92.9) | 5(100.0) |  |  |
| 2. Do you observe confidentiality? | WA | 2(1.4) | 1(1.9) | 0(0.0) | 1(2.9) | 0(0.0) | 0(0.0) |  | 0.00 |
|  | CA | 138(98.6) | 53(98.1) | 32(100.0) | 34(97.1) | 14(100.0) | 5(100.0) |  |  |
| 3. Do you obtain informed consent before testing for HIV? | WA | 116(82.9) | 45(83.3) | 24(75.0) | 29(82.9) | 13(92.9) | 5(100.0) |  | 0.5 |
|  | CA | 24(17.1) | 9(16.7) | 8(25.0) | 6(17.1) | 1(7.1) | 0(0.0) |  |  |
| 4. Is your practice influenced by the fact that you can be contaminated? | WA | 32(22.9) | 17(31.5) | 4(12.5) | 8(22.9) | 1(7.1) | 2(40.0) |  | 0.2 |
|  | CA | 108(77.1) | 37(68.5) | 28(87.5) | 27(77.1) | 13(92.9) | 3(60.0) |  |  |
| 5. Do you avoid stigmatization? | WA | 50(35.7) | 25(46.3) | 6(18.8) | 10(28.6) | 6(42.9) | 3(60.0) |  | 0.07 |
|  | CA | 90(64.3) | 29(53.7) | 26(81.3) | 25(71.4) | 8(57.1) | 2(40.0) |  |  |
| 6. Do you avoid discrimination? | WA | 41(29.3) | 25(46.3) | 5(15.6) | 8(22.9) | 2(14.3) | 1(20.0) |  | 0.02 |
|  | CA | 99(70.7) | 29(53.7) | 27(84.4) | 27(77.1) | 12(85.7) | 4(80.0) |  |  |
| 7. Do you need special motivation to take care of the infected women? | WA | 50(35.7) | 22(40.7) | 7(21.9) | 13(37.1) | 4(28.6) | 4(80.0) |  | 0.2 |
|  | CA | 90(64.3) | 32(59.3) | 25(78.1) | 22(62.9) | 10(71.4) | 1(20.0) |  |  |
| 8. Do you need special material to protect yourself? | WA | 85(60.7) | 34(63.0) | 16(50.0) | 22(62.9) | 9(64.3) | 4(80.0) |  | 0.7 |
|  | CA | 55(39.3) | 20(37.0) | 16(50.0) | 13(37.1) | 5(35.7) | 1(20.0) |  |  |
| 9. Are you interested in the newborn protection? | WA | 4(2.9) | 3(5.6) | 1(3.1) | 0(0.0) | 0(0.0) | 0(0.0) |  | 0.7 |
|  | CA | 136(97.1) | 51(94.4) | 31(96.9) | 35(100.0) | 14(100.0) | 5(100.0) |  |  |
| 10. Will you like to continue to take care of HIV infected women? | WA | 6(4.3) | 5(9.3) | 0(0.0) | 1(2.9) | 0(0.0) | 0(0.0) |  | 0.02 |
|  | CA | 134(95.7) | 49(90.7) | 32(100.0) | 34(97.1) | 14(100.0) | 5(100.0) |  |  |

NA: Nurse aide, AN: Assistant nurse, SRN: State-registered nurse, MW: Midwife, HT: Health technician, CA: Correct answer, WA: Wrong answer.
