# Supplementary material for: Knowledge, attitudes and practices of health personnel of maternities in the prevention of mother-to-child transmission of HIV in a sub-Saharan African region with high transmission rate: some solutions proposed
Source: BMC Pregnancy Childbirth. 2018 Jun 14;18:227. doi: 10.1186/s12884-018-1876-0 (PMC6000955; doi:10.1186/s12884-018-1876-0)
Supplement: Supplementary file 3 — Table S3. Practice distribution according to grade of participants. Contains details of answers assessing practice according to grade of participants as well as the statistical analysis. (DOC 56 kb) [file 12884_2018_1876_MOESM3_ESM.doc]

**Additional file 3: Table S3. Practice distribution according to grade of participants.**

| Questions Answer | | Grade of participants | | | | | | | |
| --- | --- | --- | --- | --- | --- | --- | --- | --- | --- |
|  |  | Total  N(%) | NA  N(%) | AN  N(%) | SRN  N(%) | MW  N(%) | HT  N(%) |  | P |
| 1. What do you do if the contractions are weak? | WA | 101(72.1) | 33(61.1) | 27(84.4) | 27(77.1) | 10(71.4) | 4(80.0) |  | 0.3 |
|  | CA | 39(27.9) | 21(38.9) | 5(15.6) | 8(22.9) | 4(28.6) | 1(20.0) |  |  |
| 2. What do you do in case of premature rupture of membranes lasting more than four hours? | WA | 62(44.3) | 20(37.0) | 18(56.3) | 18(51.4) | 2(14.3) | 4(80.0) |  | 0.01 |
|  | CA | 78(55.7) | 34(63.0) | 14(43.8) | 17(48.6) | 12(85.7) | 1(20.0) |  |  |
| 3. How frequently do you perform digital vaginal examination? | WA | 77(55) | 30(55.6) | 18(56.3) | 20(57.1) | 4(28.6) | 5(100.0) |  | 0.03 |
|  | CA | 63(45) | 24(44.4) | 14(43.8) | 15(42.9) | 10(71.4) | 0(0.0) |  |  |
| 4. Do you conduct artificial rupture of membranes? | WA | 29(20.7) | 14(25.9) | 1(3.1) | 10(28.6) | 3(21.4) | 1(20.0) |  | 0.08 |
|  | CA | 111(79.3) | 40(74.1) | 31(96.9) | 25(71.4) | 11(78.6) | 4(80.0) |  |  |
| 5. Do you disinfect the umbilical cord prior to its section? | WA | 75(53.6) | 29(53.7) | 15(46.9) | 20(57.1) | 7(50.0) | 4(80.0) |  | 0.8 |
|  | CA | 65(46.4) | 25(46.3) | 17(53.1) | 15(42.9) | 7(50.0) | 1(20.0) |  |  |
| 6. Do you milk the umbilical cord prior to its section? | WA | 46(32.9) | 18(33.3) | 10(31.3) | 14(40.0) | 3(21.4) | 1(20.0) |  | 0.8 |
|  | CA | 94(67.1) | 36(66.7) | 22(68.8) | 21(60.0) | 11(78.6) | 4(80.0) |  |  |
| 7. Do you systematically aspirate the newborns airways? | WA | 36(25.7) | 21(38.9) | 5(15.6) | 6(17.1) | 0(0..0) | 4(80.0) |  | 0.001 |
|  | CA | 104(74.3) | 33(61.1) | 27(84.4) | 29(82.9) | 14(100) | 1(20.0) |  |  |
| 8. Do you bathe the newborn with an antiseptic solution? | WA | 96(68.6) | 37(68.5) | 27(84.4) | 21(60.0) | 9(64.3) | 2(40.0) |  | 0.1 |
|  | CA | 44(31.4) | 17(31.5) | 5(15.6) | 14(40.0) | 5(35.7) | 3(60.0) |  |  |
| 9. Do you administer nevirapine to the newborn within the first 48 first hours? | WA | 16(11.4) | 9(16.7) | 0(0.0) | 4(11.4) | 0(0.0) | 3(60.0) |  | 0.02 |
|  | CA | 124(88.6) | 45(83.3) | 32(100) | 31(88.6) | 14(100) | 2(40.0) |  |  |
| 10. Do you screen all parturients with unknown HIV status? | WA | 6(4.3) | 2(3.7) | 1(3.1) | 2(5.7) | 1(7.1) | 0(0.0) |  | 0.9 |
|  | CA | 134(95.7) | 52(96.3) | 31(96.9) | 33(94.3) | 13(92.9) | 5(100.0) |  |  |

NA: Nurse aide, AN: Assistant nurse, SRN: State-registered nurse, MW: Midwife, HT: Health technician, CA: Correct answer, WA: Wrong answer.
